# Development of Innovative Chewable Gel Tablets Containing Nutmeg Essential Oil Microcapsules and Their Physical Properties Evaluation

**DOI:** 10.3390/pharmaceutics13060873

**Published:** 2021-06-12

**Authors:** Inga Matulyte, Mindaugas Marksa, Jurga Bernatoniene

**Affiliations:** 1 Department of Drug Technology and Social Pharmacy, Medical Academy, Lithuanian University of Health Sciences, LT-50161 Kaunas, Lithuania; inga.matulyte@lsmu.lt; 2 Institute of Pharmaceutical Technologies, Medical Academy, Lithuanian University of Health Sciences, LT-50161 Kaunas, Lithuania; 3 Department of Analytical and Toxicological Chemistry, Medical Academy, Lithuanian University of Health Sciences, LT-50161 Kaunas, Lithuania; mindaugas.marksa@lsmu.lt

**Keywords:** chewable gel tablet, nutmeg, gelatin, essential oil, microcapsule, freeze-drying, headspace, firmness, springiness, pharmaceutical research

## Abstract

Chewable gel tablets are a dosed pharmaceutical form, which can have an active substance, pharmacological effect, or value of nutrition. The texture of these tablets is soft, springy, flexible, and elastic—this is influenced by the chosen ingredients. The aim of this study was to prepare chewable gel tablets with nutmeg essential oil-loaded microcapsules and determine the volatile compounds released from this pharmaceutical form. Gel tablets were prepared by using gelatin as basis, nutmeg essential oil as active compound, and natural ingredients: thyme-sugar syrup, thyme extract, and citric acid as taste and color additives. Texture properties were measured by a texture analyzer. The release of volatile compounds from nutmeg essential oil and gel tablets were analyzed by headspace-gas chromatography with mass spectroscopy in control and artificial saliva conditions in vitro. Nutmeg essential oil microcapsules had influence on the gel tablet’s physical properties (*p* < 0.05, by comparing tablets without glycerol and relative sample with glycerol); glycerol protects the tablets from the formation of sugar crystals on top and keeps good physical parameters (*p* < 0.05). A total of 12 volatile compounds were identified in nutmeg essential oil, and the six compounds with the highest amounts were selected as controls. Gel tablets prolong the release time of volatile compounds and reduce the amount of the compounds compared to the microcapsules (*p* < 0.05).

## 1. Introduction

Essential oil is an oily liquid which has specific odors and many other effects. It is popular in aromatherapy as essential oils can reduce pain, anxiety, lift mood, suppress bacterial growth, and many other effects [1]. *Myristica fragrans* seeds are often used as a spice for taste, but they also have antibacterial and antifungal properties, and could be used as a preservative to prolong a product’s shelf-life and protect it from bacterial and fungi growth [2,3]. Nutmeg essential oil has a higher antibacterial effect compared to other products, such as aqueous extracts or hydrolats, as it is a concentrated liquid with a large amount of compounds—14–29% sabinene, 15–28% α-pinene, 13–18% β-pinene, 2–6% γ-terpinene, and other compounds—which have a wide range of antibacterial effects (compound concentration values are taken from European Pharmacopoeia) [4,5,6,7,8,9]. Nutmeg seed essential oil has antioxidant, antidepressant, antibacterial, antifungal, hepatoprotective, anti-inflammatory, and insecticidal effects, which are important in the development of pharmaceutical forms [5,10]. Essential oil is one of the most popular natural products; it has a significant effect against bacteria and could be used as a preservative [11]. Essential oils can suppress gram-negative and gram-positive bacteria. For example, nutmeg essential oil has activity against gram-negative bacteria: *Escherichia coli, Yersinia enterocolitica, Proteus spp., Klebsiella pneumoniae, Pasteurella multocida, Shigella dysenteriae,* and *Salmonella typhi*; it also can suppress gram-positive bacteria: *Streptococcus mutans, Streptococcus aureus, Streptococcus epidermis,* and *Enterococcus faecalis* [6,7,12]. In a study by D. D. Shirurkar et al. (2012), nutmeg essential oil showed promising results against fungi: *Aspergillus flavus, Aspergillus niger, Aspergillus terreus, Aspergillus oryzae, Aspergillus fumigatus, Fusarium moniliforme, Fusarium solani*, and *Penicillium* spp [13]. Essential oils and aromatic plant extracts can be used in the pharmaceutical (for medicines), cosmetic (for skin products, perfumes), and food (as preservative, flavor, fragrance) industries [11]. Essential oils can be used as active compounds, having a certain pharmacological effect.

The main limitation is instability—essential oil is labile, therefore when exposed to external factors it loses its effectiveness as the volatile compounds evaporate, spreading in the air [14]. Microcapsules made with essential oils are commonly more resistant to some conditions: acid, pH value, high temperature, and moisture, as the core of the active substance is protected by a shell [15,16,17]. Microencapsulation by lyophilization (freeze-drying) is one of the many microencapsulation methods, along with extrusion, spray-drying, and simple and complex coacervation, which can protect essential oil compounds from environmental impact [15,18,19,20]. Essential oil is a core of a microparticle (active substance) and a few other materials, such as gelatin, starch, maltodextrin, and gum arabic, which are used as a wall material to make a microcapsule [21,22,23,24]. Microcapsules prepared by freeze-drying have a shape similar to broken glass; the difference in particle morphology may be associated with shell materials as they could modify the surface [15,25]. Microcapsules may be an intermediate form that could be used in developing other forms, such as tablets [26,27].

Chewable gel tablets are tablets with a gel base, similar to gummies. However, these tablets are meant to be used in a pharmaceutical industry as drugs, food supplements, or as a medical device. Gel-based tablets are prepared by using silicones or metal forms, and, unlike simple tablets, are not compressed by tablet machine punches [28,29,30]. Palatable chewable gel tablets must have sweeteners in their composition [31,32,33]. Sweeteners can make the chewable gel tablet tastier and hide the sensory properties of other substances that are undesirable [3,28,34]. In the industry, natural, semi-natural, or chemical sweeteners are usually used. Sucrose is the most popular sweetener which is obtained from sugar beets or canes, and it is natural [35,36].

Chewable gel tablets with essential oil microcapsules are an innovative form; we found no studies regarding it to date, so this pharmaceutical form is a new area for researchers. When chewing these tablets, the active compounds are first released in the mouth, and the rest of the compounds are excreted in the stomach and intestines; this can be adapted in creating a localized effect in the oral cavity. In addition, microcapsules can prolong active substance release (depending on the excipients) and this can be adapted for systemic effect [37,38,39,40,41]. The creation of gel-based tablets with incorporated microcapsules, such as nutmeg or other essential oils, as an active compound is a new method to develop pharmaceutical forms which can be adapted for the elderly, children, or people who have swallowing disorders [42].

This research is a new and innovative study for using microcapsules with nutmeg essential oil in the preparation of chewable gel tablets and creation of a pharmaceutical form. Future research will concentrate on the adaptation of the pharmaceutical uses of these chewable gel tablets with nutmeg essential oil microcapsules.

The aim of this study was to develop innovative chewable gel tablets with natural materials used for taste and flavor, to create gel tablets with nutmeg essential oil microcapsules as active substance, to evaluate the effect of tablets ingredients on physical changes in texture, and, finally, to determine the amount of nutmeg essential oil compounds in a gel tablet by using in vitro studies.

## 2. Materials and Methods

### 2.1. Materials

Gelatin 180 bloom (Carl Roth GmbH and Co. KG, Karlsruhe, Germany) with distilled water (LUHS laboratory, Kaunas, Lithuania) was used as a chewable gel tablet basis. Glycerol (99+% vegetable origin, Chem-Lab, Zedelgem, Belgium), thyme-sugar syrup [3] (Thyme herb from A. Karvelis therapy-phytotherapy company, Švenčionys, Lithuania; distilled water from Lithuanian University of Health Sciences laboratory, Kaunas, Lithuania; and sugar from Pfeifer and Langen Marketing Sp., Poznan, Poland), and citric acid (Carl Roth GmbH and Co. KG, Karlsruhe, Germany) were used as excipients (texture, flavor, and color) in the manufacturing of chewable gel tablets. Nutmeg essential oil from *Myristica fragrans* seeds (seeds from Grenada, supplier Spaisvilė, Pašaltuonys, Lithuania), sodium alginate from brown algae (Sigma-Aldrich, Shanghai, China), and maltodextrin (Sigma-Aldrich, Saint Louis, MO, USA) were used in freeze-dried microcapsules. Magnesium aluminometasilicate (Fuji Chemical Industries Co., Ltd., Toyoma, Japan) was used as an excipient in essential oil production. Helium (99.999%, Linde, Vilnius, Lithuania) was used as carrier gas for headspace-gas chromatography with mass spectroscopy (HS-GC-MS).

Materials for artificial salivary fluid were from Carl Roth GmbH and Co. KG, Karlsruhe, Germany; all components are mentioned in “2.9. Volatile Compounds Release In Vitro” section.

### 2.2. Nutmeg Essential Oil Preparation

Nutmeg essential oil was prepared by hydrodistillation. Nutmeg seed powder was mixed with magnesium aluminometasilicate and then distilled water was added to the mixture (5:1:100) [43]. Hydrodistillation (using Clevenger-type apparatus) was carried out for 2 h. A colorless essential oil with a specific odor was obtained. It was collected in an airtight bottle and stored in a refrigerator at 4 °C, until needed.

### 2.3. Microcapsules’ Preparation by Lyophilization

First, emulsions with nutmeg essential oil were prepared using: sodium alginate, maltodextrin, water, and nutmeg essential oil. Four samples were prepared. Essential oil concentrations in the emulsion were 0%, 20%, and 25%. Sodium alginate and maltodextrin ratio ranged from 1:0 to 1:10 (Table 1).

Sodium alginate was dissolved in water, then maltodextrin was added. Stirring was carried out for 10 min and then essential oil was poured in the solution. The solution was homogenized with T18 digital Ultra Turrax^®^ (IKA^®^-Werke GmbH & Co. KG, Staufen, Germany) homogenizer for 15 min at 450 rpm. 

The emulsion was refrigerated for 48 h at −18 °C. Freeze-drying was carried out by using Beta 1–8 LSCplus lyophilizer (Martin Christ Gefriertrocknungsanlagen GmbH, Osterode am Harz, Germany). The condenser of the apparatus was switched on for approximately 30 min before the start of lyophilization to reach the required temperature (−53 °C). The emulsion was lyophilized for 48 h; afterwards the mass was ground into a small powder and stored in an airtight box.

### 2.4. Morphology of Microcapsules

Structural analysis of the freeze-dried microcapsules was conducted by scanning with electron microscope QUANTA200FEG (FEI Company, Hillsboro, OR, USA), using 100× and 500× magnification. A small amount of freeze-dried powder sample was placed on the specimen holder and images were recorded at 10 kV.

### 2.5. Microcapsules’ Physical Properties Evaluation

Microcapsules prepared by freeze-drying were evaluated by assessing moisture content, yield, and powder properties (compressibility index and Hausner ratio). 

Moisture was measured with Moisture analyzer DAB (KERN & SOHN Gmb, Balingen, Germany). A sample (0.2 ± 0.05 g) was weighed and heated (105 °C) to a constant mass, and moisture content (%) was shown in a device’s screen. 

Yield of the prepared nutmeg essential oil microcapsules was calculated. The amount of dry materials and nutmeg essential oil (theoretical value), which were used to prepare capsules was calculated, and divided from the practical amount of microcapsules (mass which was obtained after lyophilization). The moisture content of the microcapsules was taken into account in calculating the yield.

The microcapsules’ properties were evaluated by using TD 1 Tap Density Tester (SOTAX, Hopkinton, United States). Volume and amount were entered in the device. The device taps 10 times, then the changed volume is entered, and the device continues tapping for 490 times and the Hausner ratio with compressibility index is measured (values were shown in a device’s screen). All measurements were carried out three times.

### 2.6. Chewable Gel Tablets’ Preparation

The basis of the gel tablets was prepared using gelatin, water, and glycerol (ratio 1:1:1) or gelatin and water (ratio 1:2). Gelatin was poured in either distilled water, or distilled water mixed with glycerol, and swollen for 10–15 min. Gelatin mass (27% *w*/*w*) was melted, and thyme-sugar syrup (68.5% *w*/*w*) and thyme extract (2.5% *w*/*w*) were added. Then, citric acid 50% solution (2% *w*/*w*) was added. The mass was poured in silicone forms and left at a room temperature (25 ± 2 °C) for a night. Chewable gel tablets were stored in an airtight box in the dark. 

Six samples of chewable gel tablets (without essential oil—Cgt; with nutmeg essential oil—CgtN; and with nutmeg essential oil-loaded microcapsules—CgtNM; tablets with glycerol have the letter G in the abbreviation, for example, GCgtN—chewable gel tablet with essential oil and glycerol) were prepared. Nutmeg essential oil (0.469% *w*/*w*) or nutmeg essential oil microcapsules (3.75% *w*/*w*) were added in the basis of chewable gel tablets before pouring the mixture into forms. 

The selected amount of nutmeg essential oil was equal to the amount of essential oil in the microcapsules. The amount of nutmeg essential oil-loaded microcapsules was chosen by chewable gel tablet taste intensity and appearance.

### 2.7. Chewable Gel Tablets’ Physical Parameters Determination

Throughout the study, the mass of chewable gel tablets was measured and the mass variation was calculated (*n* = 10). Chewable gel tablets’ firmness, springiness, hardness, and stickiness were measured (*n* = 3) by texture analyzer Ta.XT.plus (Texture Technologies, New York, NY, USA). 

The parameters of the test for firmness and springiness were: return speed 10 mm/s; force 1 g; strain 50%; pre-test speed 1.00 mm/s; test speed 1.00 mm/s; post-test speed 10 mm/s; hold time 60 s; and trigger force 5.0 g. 

The parameters of the test for hardness and stickiness were: height of probe 20 mm; test speed 2 mm/s; and distance 5 mm (then the probe touches the tablet and the distance is measured). 

### 2.8. Nutmeg Essential Oil’s Quality and Quantity Determination

Nutmeg essential oil’s chemical compounds were determined using headspace-gas chromatography with mass spectroscopy (HS-GC-MS). A compounds’ amount was determined in nutmeg essential oil, microcapsules, and chewable gel tablets. 

For analysis, 19 ± 0.74 mg of nutmeg essential oil, 130 ± 5.34 mg of microcapsules, or one chewable gel tablet (Cgt, CgtN, and CgtNM; 3.46 ± 0.22 g) which was cut crosswise into four parts, were inserted into a 20 mL vial, which was hermetically closed with a screw cap and silicone septum. Vials with samples were kept at 40 °C for 20 min in a temperature-controlled apparatus to reach an equilibration state of the volatile compounds partition in the vapor phase of the headspace. A vapor phase was collected with a 0.5 mL gastight syringe (Kit HS syringe 2.5mL, CombiPAL, Santa Clara, CA, USA) and injected into a GCMS-QP2010 system (Shimadzu, Tokyo, Japan), syringe’s temperature was 80 °C, injection speed 500 µL/s. 

The parameters of GC-MS were: column 30 m × 0.25 i.d. × 0.25 µL film thickness RTX-MS column; oven temperature was maintained at 40 °C for 2 min after injection, then increased by 3 °C/min until 210 °C; split ratio 1:10; mass detector electron ionization was 70 eV; mass spectra library search (NIST14, NIST14s, WR10, WR10R) was used to identify volatile compounds; and the helium carrier gas flow rate was set at 1.23 mL/min.

Each experiment was triplicated, and the mean of the results are presented with standard deviation.

### 2.9. Volatile Compounds’ Release In Vitro

Volatile compounds from nutmeg essential oil-loaded sodium alginate microcapsules and chewable gel tablets with these microcapsules were determined using an in vitro study: 130 ± 5.34 mg of microcapsules or one chewable tablet (GCgtNM, 3.57 ± 0.18 g) was placed in a glass with artificial saliva, covered, and then stirred in a magnetic stirrer at 37 °C. Samples (1 mL of solution) were taken after 5, 15, 30, and 60 min, poured in a bottle, and analyzed with HS-GC-MS. Conditions are described in “2.7. Nutmeg Essential Oil’s Quality and Quantity Determination” section. 

Artificial salivary fluid with pH = 6.8 value was prepared; the composition is given in Table 2 [44].

### 2.10. Statistical Analysis

Data are presented as the mean ± SD. Statistical analysis was performed with SPSS (IBM Corporation, New York, NY, USA). One-way ANOVA was used to investigate the differences between physical parameters of tablets and volatile compounds. Post hoc comparisons of the means were performed according to Turkey’s HSD test. The results were significant when *p* < 0.05.

## 3. Results and Discussion

### 3.1. Preparation and Evaluation of Microcapsules with Nutmeg Essential Oil

Nutmeg essential oil was chosen as an active pharmaceutical ingredient for this study. It has a wide range of effects which can be adapted in a pharmacological use [2,7,45]. *Myristica fragrans* seed essential oil has about 7% higher antioxidant activity than *Juniperus scopulotum* (composition of compounds was similar) [7,46]. Volatile compounds from nutmeg essential oil (0.5%) are effective against bacteria (*E. faecalis*) of which growth in the oral cavity is increased after dental procedures [47]. In a previous study, it was determined that nutmeg essential oil has preservative properties, and can significantly increase the shelf-life of chewable gel tablets compared to sodium benzoate or citric acid (176, 75, and 108 days, respectively) [3].

Nutmeg essential oil was prepared by using hydrodistillation. It was a colorless oily liquid. The excipient in hydrodistillation was used as magnesium aluminometasilicate significantly increases some of the volatile compounds’ amount and the essential oil yield (from 5.25 ± 0.04% to 10.43 ± 0.09%) [43]. 

Nutmeg essential oil microcapsules were prepared by lyophilization; four different samples of microcapsules were manufactured (microcapsules’ composition is presented in Table 1). 

**Figure 1:**
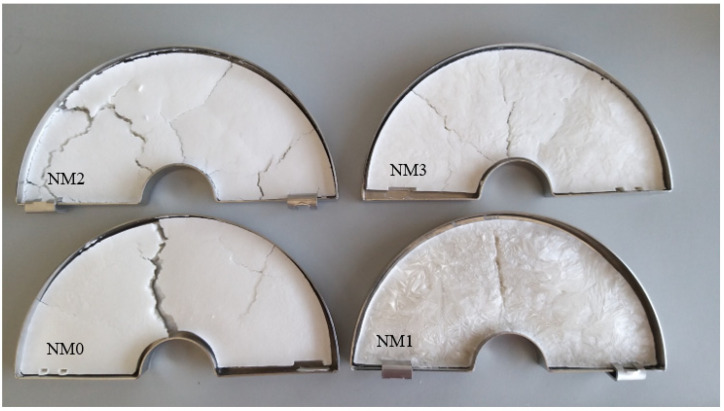
Freeze-dried nutmeg essential oil microcapsules. Codes’ meaning is provided in Table 1.

Microcapsules NM0 were chosen as a control group; the other three samples were prepared with nutmeg essential oil. The powder properties of the microcapsules from all the samples were determined. NM1 microcapsules’ texture was similar to polystyrene foam, and it was impossible to mesh it out; NM2 and NM3 microcapsules were easy to pulverize. All samples with nutmeg essential oil had a specific odor. The view of microcapsules’ mass after freeze-drying is shown in Figure 1.

Microcapsules with essential oil (NM2 and NM3) were a white colored powder. Mass with a higher amount of oil had a less smooth surface than NM2. NM1 sample’s color was opalescent white, texture was soft but not smooth.

While determining physical parameters of microcapsules with nutmeg essential oil (Table 3), it was observed that NM1 had a higher yield of microcapsules; these microcapsules had a higher moisture content, and sodium alginate had an influence on the structure of these powders. Sample NM0 was used as control so the essential oil’s influence on physical parameters of microcapsules could be evaluated.

In this study, maltodextrin and sodium alginate were chosen as carriers. Maltodextrin is usually used in freeze-drying as a good carrier for plant extracts and essential oils [48,49]. Studies using sodium alginate as a shell material for freeze-drying were not found, however some studies used sodium alginate in spray-drying [50]. Sodium alginate has poor emulsifier properties, but higher amounts can emulsify oils. Some excipients (maltodextrin) were also used. 

In other studies, the amount of essential oil needed for freeze-drying varies from 5% to 20%, but the shell materials differed (gum arabic, starch, and gelatin) [23,51]. The yield of microcapsules was higher than 70% but lower than 82% [51]. In this study, the yield of nutmeg microcapsules was not lower than 84.03 ± 3.08%. When the yield of microcapsules is low, it can be assumed that the essential oil evaporates. The yield of microcapsules was higher when extracts were used in microencapsulation as they decrease evaporation [25]. Microcapsules with oregano essential oil prepared by spray-drying had 3.4–3.7 ± 0.2% moisture (essential oil amount in microcapsules was from 3% to 6%) [52]. These results show that nearly 50% of oregano essential oil evaporated.

**Figure 2:**
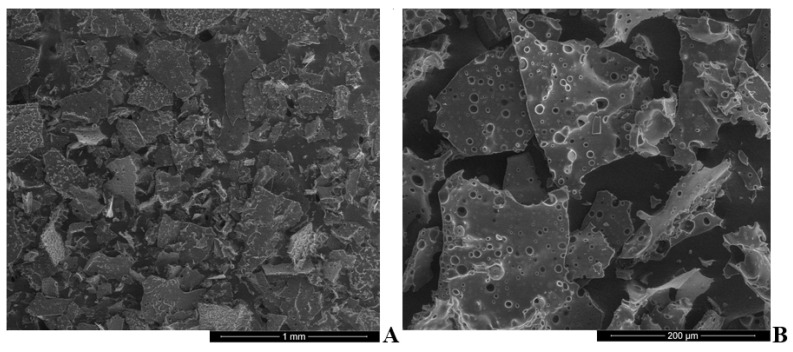
Microstructure of freeze-dried NM3 microcapsules, (**A**) 100×, and (**B**) 500× magnification.

All powders (Table 3) had passable or poor flow determined by compressibility index (the index’s values are presented in monograph of European Pharmacopoeia [53]). The moisture content in NM1 sample was the highest; sodium alginate with essential oil was not completely dried during freeze-drying process. These powders had the worst parameters compared with other samples. Although the moisture content exceeds the content of the essential oil, this mass is not suitable for use as microcapsules. For further research, the NM3 sample with 25% of essential oil was chosen—although the yield is lower than NM2, the powders (NM3) have better characteristics, and they are easier to incorporate into a pharmaceutical form. The morphology of NM3 powder is shown in Figure 2.

Images of freeze-dried microcapsule powder presented an irregular shape, like broken glass, with some essential oil pores on surface. NM3 microcapsules were used for the manufacture of chewable gel tablets.

### 3.2. Preparation and Physical Properties Evaluation of Chewable Gel Tablets with Nutmeg Essential Oil

Chewable gel tablets were made with thyme extract as a flavor and color additive, and gelatin, which formed a chewable tablet. Gelatin is produced by partial hydrolysis of native collagen and is usually used in the food, medicine, and cosmetic industries as a thickener, capsule ingredient, and as a protein source [54,55,56]. Gelatin was used to form chewable gel tablets with nutmeg essential oil and microcapsules. In other studies, gelatin’s amount varies from 6.5% to 15.38% [3,57,58,59]. 

**Figure 3:**
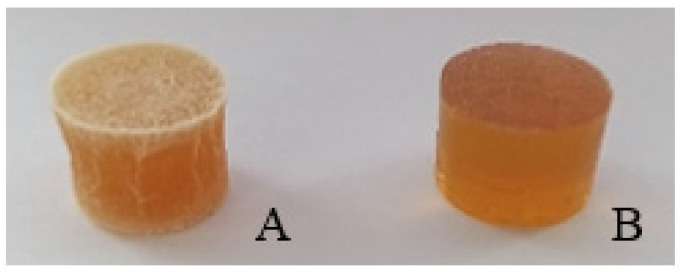
Chewable gel tablets’ visual texture differences between tablets without glycerol (**A**) and with glycerol (**B**) in composition.

Six compositions of tablets were prepared (code meaning is given in “2.6. Chewable Gel Tablets’ Preparation” section), and glycerol’s influence on tablets’ properties was determined. A visual difference between gel tablets with and without glycerol is shown in Figure 3.

A chewable gel tablet without glycerol (Figure 3A) is harder and has crystallized sugar on top. The tablet was difficult to compress, and the surface cracks when pressed. A tablet with glycerol is elastic, glossy, and translucent (Figure 3B). 

**Figure 4:**
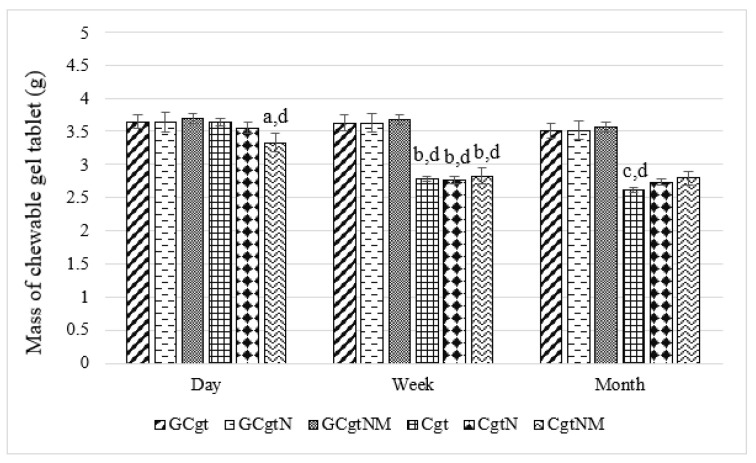
Mass variation in different chewable gel tablets, *n* = 10; (**a**) *p* < 0.05 versus all samples with glycerol (GCtg, GCtgN, and GCtgNM); (**b**) *p* < 0.05 versus relative samples after one day; (**c**) *p* < 0.05 versus Cgt sample after one week; and (**d**) *p* < 0.05 versus relative samples with glycerol in composition; code meaning is given in “2.6. Chewable Gel Tablets’ Preparation” section.

In further study, the physical properties of the created tablets were evaluated. All chewable gel tablets, (tablets with glycerol, which were protected from sugar crystallization, and samples without glycerol) were weighed and mass variation was measured. Measurements were taken one day, week, and month after the production of gel tablets (Figure 4). 

In tablets with glycerol, statistical significance of mass variation was not found throughout the study period. The CtgNM sample’s mass was significantly lower compared with samples which had glycerol in composition. Microcapsules with essential oil increased the volume of a gel tablet, but not the mass. The mass of samples with essential oil (CgtN) was a little lower, but this may have been due to the evaporation of the essential oil. After one week, all samples without glycerol had a lower mass. About 20.26% of weight was lost, *p* < 0.05. Cgt sample’s mass average after one month was 6.11% lower than after a week, *p* < 0.05 (Figure 4). In other studies, gel tablets without glycerol lost 44.08–50.95% of the mass (tablets were kept in room temperature and an opened box) [3]. 

Glycerol protects pharmaceutical forms from water loss and sugar crystallization; this is evidenced by the tablet in Figure 3. Other properties of glycerol mentioned in articles are that: it is one of the most effective humectant polyols used in food; it has a significant moisturization effect; it is nontoxic; it provides a good flavor and a pleasant odor; and it protects from sugar crystallization [60,61,62,63]. Glycerol, as an excipient in a chewable gel tablet, protects the chewable gel tablets from sugar crystallization and mass loss. 

When firmness, hardness, stickiness, and springiness was compared in six samples of chewable gel tablets with nutmeg products, it was determined that glycerol has a significant influence on these properties (Table 4).

**Figure 5:**
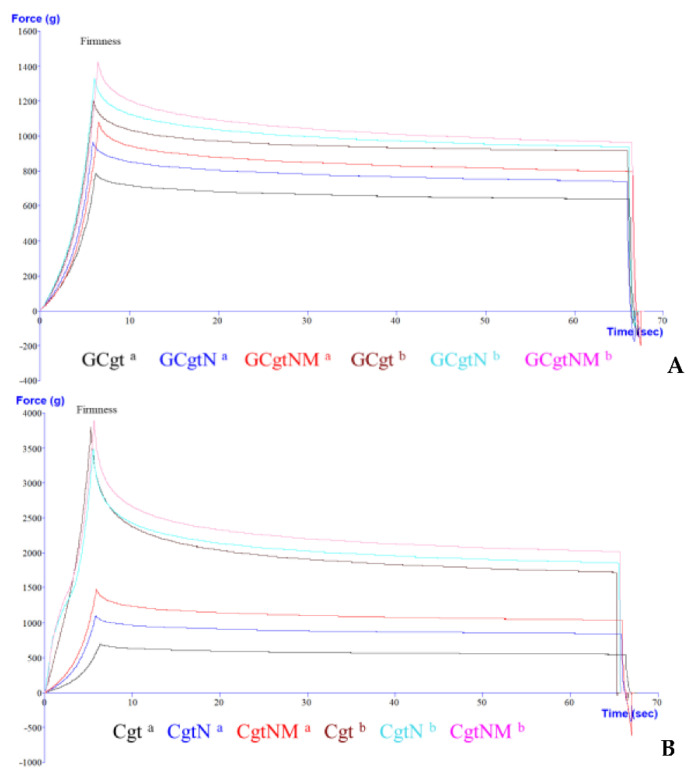
Firmness and springiness graph of chewable gel tablets with (**A**) and without (**B**) glycerol; a—after one day, b—after one month; sample name meaning is given in “2.5. Chewable Gel Tablets Preparation” section.

**Figure 6:**
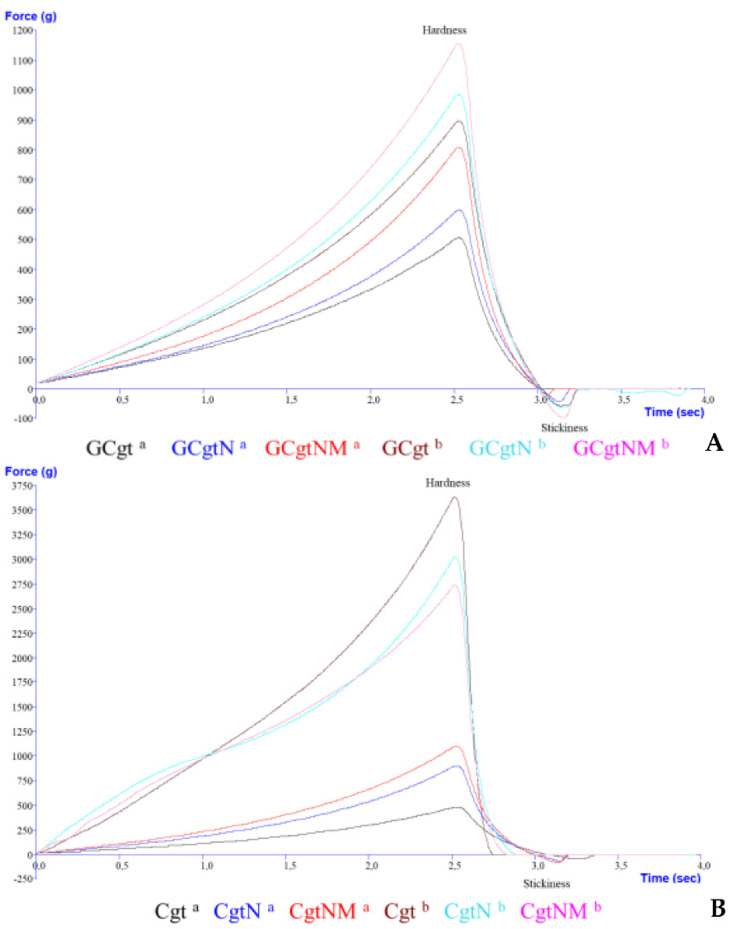
Hardness and stickiness graph of chewable gel tablets with (**A**) and without (**B**) glycerol; a—after one day, b—after one month; sample name meaning is given in “2.5. Chewable Gel Tablets Preparation” section.

To measure firmness and springiness, the probe’s distance was 5 mm from top to bottom of the chewable gel tablet. Hardness and stickiness were measured to compress 50% of a gel tablet (distance of one-day-old tablets was approximately 6.23 mm, after one month it was approximately 5.65 mm—the distance average needed to compress the tablet). Sample graphs of texture analysis (changes after one day and one month) are presented in Figure 5 and Figure 6.

Tablet samples with glycerol (GCgt, GCgtN, and GCgtNM) had lower firmness and hardness in all time periods compared to tablets without glycerol (Table 4). After one month, these parameters were 1.33–1.53 and 1.42–1.64 times higher (firmness and hardness, respectively) in samples with glycerol, *p* < 0.05. Nutmeg essential oil microcapsules had a higher influence on chewable gel tablets—they reduced the effect of these parameters and the tablets hardened the least; tablets without additive compounds (nutmeg essential oil and microcapsules) were the hardest. In other studies, a few parameters of gummies were evaluated, and it was determined that 0.2% of cloves or thyme essential oils decreased hardness about 1.59 and 1.86 times, respectively [64].

Chewable gel tablets without glycerol had significant firmness and hardness parameters. After a month, 2883.81–3746.06 g of force was needed to compress the 5 mm distance of a tablet to measure the hardness parameter. Additionally, the tablets with microcapsules had lower changes in texture properties (about 2.61 times), and Cgt firmness increased 5.35, while hardness increased 7.47 times, *p* < 0.05. 

Glycerol’s influence on springiness was determined. Tablets without glycerol were less elastic. This parameter was changed by about 41.69%—Cgt, CgtN, and CgtNM lost two-fifths of their former elasticity, while tablets with glycerol lost about 7.13% of springiness, *p* < 0.05. Regarding nutmeg essential oil and nutmeg essential oil microcapsules’ influence on this parameter, it was determined that chewable gel tablets without these materials had the highest elasticity. In other studies it was determined that gel tablets with glycerol had over than 6500 g of firmness after one month [3]. 

Assessing the adhesiveness parameter (stickiness), it was established that chewable gel tablets with glycerol have good adhesion, from −95.07 ± 8.28 to −76.90 ± 6.66 g, while tablets without glycerol had from −1.38 ± 1.05 to −0.12 ± 0.04 g of stickiness. These values show that the higher value of adhesiveness implies a softer texture [65]. Stickiness is defined as the negative force area (measured in grams) for the first bite, and represents the work required to overcome the attractive forces between the surface of a product and the surface of other materials with which the product comes into contact [65,66].

The results of this study have shown that to prepare a palatable and suitable (i.e., physical parameters changing the least) chewable gel tablet, glycerol is required as a moisture agent, and some other additives, which provide powder consistency, should be used as well. Freeze-dried powders (microcapsules) with nutmeg essential oil could protect the tablets from hardening. Glycerol increases elasticity and improves adhesion of chewable gel tablets.

### 3.3. Volatile Compounds Evaluation of Chewable Gel Tablets Containing Nutmeg Essential Oil 

Gelatin gel melts in 10–40 °C temperatures, depending on the amount and type of gelatin used. The gelatin obtained from fish has a lower melting temperature than the mammalian gelatin [67,68]. In this study, mammalian gelatin was used, and the temperature required for volatile compounds to be released from gel tablets was 40 °C. 

**Figure 7:**
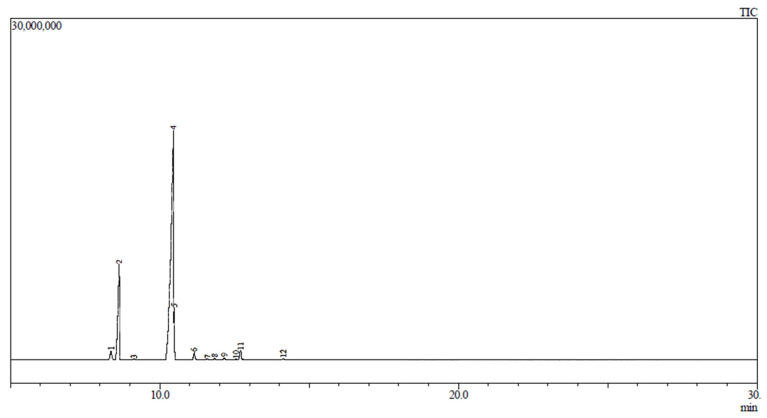
HS-GC-MS chromatogram of nutmeg essential oil.

For this part of the study, chewable gel tablets with glycerol were chosen as their physical properties were evaluated as appropriate. These tablets did not have sugar on top, which could have influence on the volatile compounds’ release, and their texture was good—not too solid. Volatile compounds were identified in nutmeg essential oil and in microcapsules by using HS-GC-MS; three samples of chewable gel tablets were also evaluated (GCgt, GCgtN, and GCgtNM). In an empty gel tablet, without nutmeg essential oil (GCgt), not a single compound was identified. A chromatogram of nutmeg essential oil is presented in Figure 7 (all identified compounds).

In nutmeg essential oil, 12 chemical compounds were identified, whereas six of these compounds were also found in chewable gel tablets or microcapsules (compounds with the highest amounts were selected as control markers). It was determined that Sabinene and beta Ocimene were present in the highest amounts in the essential oil (Table 5). Nutmeg essential oil was prepared by using a modified hydrodistillation, and magnesium aluminometasilicate was also used. This material statistically significantly increases some of the volatile compounds’ (Sabinene and Limonene) and essential oil’s yield. Nutmeg essential oil was analyzed by a gas chromatography-mass spectroscopy method and 21–24 volatile compounds were determined (the highest amounts were of Sabinene and α-Pinene, 61.42% and 15.05%, respectively) [43]. 

Nutmeg essential oil microcapsules and gel tablets released a lower number of volatile compounds. In microcapsules and chewable gel tablets with essential oil, nine compounds were identified; in gel tablets with microcapsules, only 5 compounds were identified.

Using HS-GC-MS analysis to determine volatile compounds, temperature, and time, which are needed to reach the equilibration state of the volatile compounds partition in the vapor phase, can influence the amount of identified volatile compounds [69].

### 3.4. Volatile Compounds Release In Vitro

Human saliva pH is between 5.8 and 7.3, and varies depending on the secretory activity of the glands. The main component of saliva is water (up to 99.5%), and the level of inorganic compounds ranges from 0.2 to 0.9%, while an organic fraction ranges from 0.4 to 0.6% [70]. In this study, we used artificial saliva fluid (composition can be found in Table 2). 

Volatile compounds were determined in microcapsules with nutmeg essential oil and in chewable gel tablets containing nutmeg essential oil-loaded microcapsules. Results of the compounds release from microcapsules are presented in Table 6 and Table 7. The amount of volatile compounds from microcapsules loaded with nutmeg essential oil was calculated using the amount of essential oil compounds (the quantity of essential oil and oil in microcapsules had a 5% error). 

The number of compounds was calculated from the number of compounds released in the nutmeg essential oil.

In microcapsules with nutmeg essential oil, all six compounds that were identified in essential oil were determined. After 5 min in artificial saliva (microcapsules dissolve in the solution after 3 min), the best released compounds were: Limonene—approximately 60%; beta Myrcene—approximately 36%; and beta Pinene—approximately 30%. Other results after 15 min and longer were significantly lower; this is due to the fact that these compounds are volatile, meaning they can be exposed as the temperature was 37 °C degrees, therefore until they are transferred to the vial, they evaporate. The maximum number of compounds released after 5 min indicates the quality of the produced microcapsules, therefore they can be used in the manufacture of chewable gel tablets. The greatest amount is released after the shortest time, and then decreases; the remaining compounds will be released in the stomach and intestines [71]. 

In other studies with thyme and coriander essential oil, it was determined that 43% of volatile compounds are released from microcapsules, and the highest amount of the release of volatile compounds was achieved at 120 min (the hydrolyzed collagen was a shell) [72]. Lemongrass essential oil relaxation from microcapsules with chitosan ranged from 2.5 ± 0.2% to 78.06 ± 8.7% [73]. 

To determine active compound release from chewable gel tablets with nutmeg essential oil-loaded microcapsules, the amount was calculated from the amount released in the microcapsules (Table 7).

Chewable gel tablets dissolved after 9 min in an artificial saliva fluid, and this property had an influence on the release of chemical compounds. In these tablets, only four compounds were identified; beta Pinene and beta Myrcene were not found in the gel tablets after in vitro study. Gelatin had an influence on the compounds’ relaxation. The highest amount was determined in the saliva fluid after 30min. All results were statistically significant compared to a different time sample. 

As the chewable gel tablet was cut into only four parts, this affected the dissolution of the gelatin. When the gelatin first dissolved, the alginate capsules, which are soluble in an alkaline medium, could also be dissolved. When sodium alginate microcapsules are swallowed in an alkaline medium, they dissolve, and in an acid medium swelling does not occur [20,50,74,75]. Additionally, the citric acid in the tablet reduced their dissolution, therefore it took longer for the compounds to relax. This could be avoided if the tablet was crushed more (i.e., chewed more than twice, so the tablet is crushed into more than four parts).

Limonene, Pinene, Myrcene, Ocimene, and Sabinene are monoterpenes which are found in essential oils. They have therapeutic antiseptic, bacterial, and antiviral properties, and they may also have analgesic, expectorant, decongestant, and stimulant properties as well [76]. Limonene and beta Ocimene were best released from gel tablets containing nutmeg essential oil-loaded microcapsules at over 75%. Both of these compounds have a pleasant odor, so chewing this gel tablet will result in a good taste [77,78]. 

By combining the effects of thyme syrup and nutmeg essential oil, these gel tablets could be used as a pharmaceutical form to reduce the symptoms of a common cold. Due to the presence of antibacterial and antiseptic therapeutic components of the essential oil, and the long-known positive effects of thyme extract, these tablets could be used as an expectorant and antibacterial remedy.

## 4. Conclusions

Chewable gel tablets with essential oil microcapsules were created and evaluated for the first time. It was found that glycerol’s amount has a significant impact, reducing firmness and hardness, and allowing the tablets to retain elasticity after a long time. A ration of 1:1:1 (water:glycerol:gelatin) in gel tablets protects them from sugar crystallization. After one month from when the tablets were prepared, glycerol helps maintain a higher elasticity—on average 39.22%—compared to glycerol-free chewable gel tablets (*p* < 0.05). The stickiness of tablets without glycerol was determined at a value of nearly zero, showing that these tablets have a hard texture (*p* < 0.05).

When comparing nutmeg essential oil products’ influence on chewable gel tablets, it was determined that nutmeg essential oil has a lesser influence than microcapsules with essential oil. Nutmeg essential oil-loaded sodium alginate microcapsules reduce springiness and increase firmness, hardness, and stickiness (1.05, 1.20, 1.17, and 1.24 times, respectively; results taken after one month) compared to tablets with nutmeg essential oil—all data, except springiness, are statistically significant.

The maximum amount of released compounds from the microcapsules loaded with nutmeg essential oil is reached after 5 min; more than 30% of beta Pinene, beta Myrcene, and Limonene is released. Later, the amount of all compounds decreases, and the differences were statistically significant after 15 min. In a chewable gel tablet containing nutmeg essential oil-loaded microcapsules, four volatile compounds were identified, but a higher amount was determined after 30 min. Gel structure had a significant influence on the release of active substances.

This study shows that different structures of a pharmaceutical form (microcapsules and gel tablets) had an influence on volatile compounds’ release; they were much better released from microcapsules than from chewable gel tablets (*p* < 0.05). These data are basis for further research with gel tablets containing essential oil microcapsules. 

## Figures and Tables

**Table 1:** Nutmeg essential oil microcapsules’ composition.

Materials	Sample Names			
	NM0	NM1	NM2	NM3
Sodium alginate (g)	1.82	3.33	2.67	2.5
Maltodextrin (g)	18.18	-	13.33	12.5
Nutmeg essential oil (g)	-	0.832	4.0	5.0
Distilled water (mL)	60.0	60.0	60.0	60.0
Amount (%) of nutmeg essential oil in microcapsules (calculated by dry materials)	0	20.0	20.0	25.0

**Table 2:** Artificial saliva solution’s composition.

Ingredient	Concentration (mg/L)
Magnesium chloride, anhydrous	100
Calcium chloride, dihydrate	220
Sodium phosphate dibasic, heptahydrate	1350
Potassium phosphate monobasic	680
Potassium chloride	750
Urea	600
Sodium chloride	600
Distillated water	up to 1 L

**Table 3:** Physical parameters of nutmeg essential oil-loaded microcapsules, *n* = 3.

Sample	Moisture (%)	Yield (%)	Compressibility Index	Hausner Ratio
NM0	13.10 ± 2.67	88.23 ± 3.56	30.13 ± 2.14	1.429 ± 0.2
NM1	25.90 ± 2.37	115.57 ± 10.53	39.30 ± 0.57	1.503 ± 0.1
NM2	18.38 ± 0.90	92.39 ± 2.41	25.69 ± 1.42	1.346 ± 0.1
NM3	18.13 ± 2.71	84.30 ± 3.08	20.43 ± 1.63	1.257 ± 0.1

**Table 4:** Chewable gel tablets texture properties depending on the composition and time of manufacture.

Sample Name ^a^	Firmness (g)			Springiness (%)			Hardness (g)			Stickiness (g)		
	after One Day	after One Week	after One Month	after One Day	after One Week	after One Month	after One Day	after One Week	after One Month	after One Day	after One Week	after One Month
**GCgt**	798.71 ± 22.68	961.64 ± 51.48	1227.87 ± 34.20	80.46 ± 0.36	78.76 ± 1.34	75.01 ± 0.45	559.66 ± 60.27	692.10 ± 84.92	920.17 ± 116.18	−40.83 ± 0.64	−87.30 ± 7.44	−81.87 ± 20.13
**GCgtN**	892.53 ± 40.36	1052.16 ± 46.17	1251.03 ± 85.27	78.29 ± 1.32	75.64 ± 1.20	72.50 ± 2.20	581.94 ± 66.99	702.55 ± 78.29	864.03 ± 83.88	−24.90 ± 11.78	−65.92 ± 7.08	−76.90 ± 6.66
**GCgtNM**	1127.01 ± 3.18	1306.25 ± 41.31	1499.06 ± 116.83	72.32 ± 0.88	72.04 ± 1.06	67.09 ± 3.30	713.35 ± 66.93	851.17 ± 98.64	1012.23 ± 104.02	−14.50 ± 7.17	−72.47 ± 8.18	−95.07 ± 8.25
Cgt *	700.38 ± 12.66	1847.83 ± 129.18	3745.30 ± 92.53	78.07 ± 1.04	54.65 ± 0.85	45.84 ± 0.20	501.44 ± 18.24	1809.59 ± 174.26	3746.06 ± 132.05	−48.10 ± 4.13	−0.20 ± 0.07	−1.38 ± 1.05
CgtN *	1141.58 ± 28.49	2686.79 ± 67.82	2986.34 ± 34.37	74.93 ± 1.21	55.20 ± 0.37	43.31 ± 1.36	905.53 ± 64.22	2425.96 ± 118.37	2615.56 ± 218.26	−53.85 ± 17.27	−101.94 ± 58.10	−0.16 ± 0.03
CgtNM *	1407.70 ± 51.07	3077.47 ± 134.24	3708.89 ± 220.61	70.63 ± 0.69	53.15 ± 1.03	41.25 ± 0.60	1104.62 ± 101.79	2681.87 ± 111.10	2883.81 ± 166.83	−93.37 ± 5.27	−104.89 ± 45.11	−0.12 ± 0.04

* all data is *p* < 0.05 versus relative samples with glycerol in composition after one month; ^a^—sample name meaning is given in “2.5. Chewable Gel Tablets Preparation” section; *n* = 3.

**Table 5:** Nutmeg essential oil’s composition identified by HS-GC-MS.

No.	Compound Name	RI ^a^	Amount %
1	5-methyl-1,3,5-heptatriene ^b^	912	1.8
2	beta Ocimene ^b^	917	18.04
3	Camphene	925	0.19
4	Sabinene ^b^	949	73.24
5	beta Pinene ^b^	950	2.98
6	beta Myrcene ^b^	962	0.99
7	alpha Phellandrene	970	0.19
8	alpha Ocimene	974	0.23
9	alpha Terpinene	980	0.32
10	4-cymene	987	0.1
11	Limonene ^b^	990	1.69
12	gamma Terpinene	1013	0.25

^a^—retention indices by using RTX-5MS column and *n*-alkanes (C9-C22) as references; ^b^—the highest number of compounds selected as markers in microcapsules and tablets samples in an in vitro study. Study was repeated three times, and the average of the amount is not higher than 5%.

**Table 6:** Release and amount (%) of compounds in an in vitro study from microcapsules with nutmeg essential oil.

Compound Name	Time			
	after 5 min.	after 15 min.	after 30 min.	after 60 min.
5-methyl-1,3,5-heptatriene	11.72 ± 0.59	7.75 ± 0.39	6.26 ± 0.31	6.08 ± 0.30
beta Ocimene	10.61 ± 0.53	7.73 ± 0.38	6.44 ± 0.32	6.45 ± 0.32
Sabinene	19.79 ± 0.99	14.29 ± 0.71	11.28 ± 0.53	10.11 ± 0.51
beta Pinene	30.33 ± 1.52	23.06 ± 1.15	17.72 ± 0.89	20.81 ± 1.04
beta Myrcene	36.24 ± 1.81	11.36 ± 0.57	7.08 ± 0.35	6.56 ± 0.33
Limonene	60.08 ± 3.00	36.71 ± 1.83	30.48 ± 1.52	28.75 ± 1.44

**Table 7:** Release and amount (%) of compounds in an in vitro study from chewable gel tablet with nutmeg essential oil-loaded microcapsules.

Compound Name	Time			
	after 5 min.	after 15 min.	after 30 min.	after 60 min.
5-methyl-1,3,5-heptatriene	14.33 ± 0.72	51.02 ± 2.55	83.94 ± 4.19	27.60 ± 1.38
beta Ocimene	20.51 ± 1.03	70.28 ± 3.51	80.23 ± 4.01	49.33 ± 2.47
Sabinene	3.53 ± 0.18	11.49 ± 0.57	14.70 ± 0.74	8.61 ± 0.43
beta Pinene	-	-	-	-
beta Myrcene	-	-	-	-
Limonene	17.82 ± 0.89	64.25 ± 3.21	75.57 ± 3.78	40.29 ± 2.01

## Data Availability

The data presented in this study are available on request from the authors.
